# Supplementary material for: A Benefit-Cost Analysis of BackTrack, a Multi-Component, Community-Based Intervention for High-Risk Young People in a Rural Australian Setting
Source: Int J Environ Res Public Health. 2022 Aug 18;19(16):10273. doi: 10.3390/ijerph191610273 (PMC9408498; doi:10.3390/ijerph191610273)
Supplement: Supplementary file 1 [file ijerph-19-10273-s001.zip › ijerph-1797165-supplementary.pdf]

Supplementary Table S1: Costs and benefits of BackTrack by year of analysis.

	Mar-12 to Feb-13	Mar-13 to Feb-14	Mar-14 to Feb-15	Mar-15 to Feb-16	Mar-16 to Feb-17	Mar-17 to Feb-18	Mar-18 to Feb-19	Mar-19 to Feb-20	Mar-20 to Feb-21
**Costs**									
Infrastructure & Equipment	$24,390	$51,025	$46,933	$58,509	$46,641				
Operating costs (Labour)	$273,833	$493,882	$596,317	$688,619	$512,297				
Operating costs (Non-labour)	$87,436	$130,005	$176,799	$281,791	$194,475				
Additional health service costs	$0	$975	$6,824	$11,699	$8,774	$1,950	$2,437	$2,925	
Administration of tax transfers	$2,825	$3,183	$1,667	$2,188	$899				
**Total costs**	$388,484	$679,070	$828,541	$1,042,807	$763,086	$1,950	$2,437	$2,925	
**Total costs** (discounted)	$388,484	$634,645	$723,680	$851,241	$582,155	$1,390	$1,624	$1,821	
**Benefits**									
Program Income	$34,230	$67,913	$76,811	$93,382	$33,838				
Education/Training - Job training, literacy & numeracy		$4,677	$32,738	$56,121	$42,091	$9,354	$11,692	$14,030	
Education/Training - High school				$260,923	$334,026	$125,213	$62,607	$36,551	$62,607
Education/Training - Vocational further education				$88,732	$88,732			$44,366	
Physical health - Engagement with health services			$41,076	$70,415	$52,811	$11,736	$14,670	$17,604	
Homelessness & housing				$93,176	$46,588				
Employment - Increased productivity			$1,198,201	$599,100	$1,903,824	$299,550	$299,550	$652,362	
Local Government - Infrastructure vandalism (savings)	$33,666	$33,666	$33,666	$33,666	$33,666				
Crime	$12,929	$99,683	$229,995	$346,570	$450,857	$88,332			
**Total Benefits**	$80,825	$205,939	$1,612,486	$1,642,087	$2,986,434	$534,185	$388,519	$764,913	$62,607
**Total Benefits** (disc.)	**$80,825**	**$192,466**	**1,408,408**	**$1,340,432**	**$2,278,336**	**$380,867**	**$258,886**	**$476,350**	**$36,438**
**Net Social Benefit**	-$307,659	-$473,131	$783,946	$599,280	$2,223,347	$532,235	$386,081	$761,989	$62,607
**Net Social Benefit** (disc.)	**-$307,659**	**-$442,179**	**$684,729**	**$489,191**	**$1,696,181**	**$379,476**	**$257,262**	**$474,528**	**$36,438**
